# Plasma metabolomic profile is near-normal in people with HIV on long-term suppressive antiretroviral therapy

**DOI:** 10.3389/fcimb.2024.1340610

**Published:** 2024-03-14

**Authors:** Ana Virseda-Berdices, Rubén Martín-Escolano, Juan Berenguer, Juan González-García, Oscar Brochado-Kith, David Rojo, Amanda Fernández-Rodríguez, Leire Pérez-Latorre, Victor Hontañón, Coral Barbas, Salvador Resino, María Ángeles Jiménez-Sousa

**Affiliations:** ^1^ Unidad de Infección Viral e Inmunidad, Centro Nacional de Microbiología (CNM), Instituto de Salud Carlos III (ISCIII), Madrid, Spain; ^2^ Centro de Investigación Biomédica en Red en Enfermedades Infecciosas (CIBERINFEC), Instituto de Salud Carlos III (ISCIII), Madrid, Spain; ^3^ Unidad de Enfermedades Infecciosas/VIH, Hospital General Universitario “Gregorio Marañón”, Madrid, Spain; ^4^ Instituto de Investigación Sanitaria Gregorio Marañón (IiSGM), Madrid, Spain; ^5^ Servicio de Medicina Interna-Unidad de VIH, Hospital Universitario La Paz, Madrid, Spain; ^6^ Instituto de Investigación Sanitaria La Paz (IdiPAZ), Madrid, Spain; ^7^ Centre of Metabolomics and Bioanalysis (CEMBIO), Facultad de Farmacia, Universidad San Pablo-CEU, CEU Universities, Boadilla del Monte, Spain

**Keywords:** antiretroviral therapy, CD4^+^/CD8^+^ ratio, HIV, inflammation, metabolomics

## Abstract

**Background:**

Combination antiretroviral therapy (ART) has transformed human immunodeficiency virus (HIV) infection in people with HIV (PWH). However, a chronic state of immune activation and inflammation is maintained despite achieving HIV suppression and satisfactory immunological recovery. We aimed to determine whether the plasma metabolomic profile of PWH on long-term suppressive ART and immunologically recovered approximates the normality by comparison with healthy controls with similar age and gender.

**Methods:**

We carried out a cross-sectional study in 17 PWH on long-term ART (HIV-RNA <50 copies/mL, CD4^+^ ≥500 cells/mm^3^, and CD4^+^/CD8^+^ ≥1) and 19 healthy controls with similar age and gender. Metabolomics analysis was performed by gas chromatography-mass spectrometry (GC-MS) and liquid chromatography-mass spectrometry (LC-MS). The statistical association analysis was performed by principal component analysis (PCA), partial least squares discriminant analysis (PLS-DA), and Generalized Linear Models (GLM) with a gamma distribution (log-link). Significance levels (p-value) were corrected for multiple testing (q-value).

**Results:**

PCA and PLS-DA analyses found no relevant differences between groups. Adjusted GLM showed 14 significant features (q-value<0.20), of which only three could be identified: lysophosphatidylcholine (LysoPC) (22:6) (q-value=0.148), lysophosphatidylethanolamine (LysoPE) (22:6) (q-value=0.050) and hydroperoxy-octadecatrienoic acid (HpOTrE)/dihydroperoxy-octadecatrienoic acid (DiHOTrE)/epoxy-octadecadienoic acid (EpODE) (q-value=0.136). These significant identified metabolites were directly correlated to plasma inflammatory biomarkers in PWH and negatively correlated in healthy controls.

**Conclusion:**

PWH on long-term ART have a metabolomic profile that is almost normal compared to healthy controls. Nevertheless, residual metabolic alterations linked to inflammatory biomarkers persist, which could favor the development of age-related comorbidities among this population.

## Introduction

People with human immunodeficiency virus (HIV) (PWH) on combination antiretroviral therapy (ART) usually achieve undetectable viral load and CD4^+^ ≥500 cells/mm^3^, decreasing the risk of developing acquired immune deficiency syndrome (AIDS)-related events, mortality, and transforming HIV infection into a chronic disease (Zicari et al., 2019). However, ART does not eradicate HIV from the body, promoting a chronic state of immune activation and inflammation that leads to the development of non-AIDS comorbidities despite viral suppression (Zicari et al., 2019). Besides, PWH on suppressive ART generally presents persistently elevated CD8^+^ T cell counts and a low CD4^+^/CD8^+^ ratio, linked to a higher immune dysfunction (immune activation, inflammation, immunosenescence, among others), viral reservoir size, aging, comorbidities, and mortality (Lu et al., 2015; Zicari et al., 2019; Chen et al., 2022). Therefore, besides CD4^+^ ≥500 cells/mm^3^, CD4^+^/CD8^+^ ratio ≥1 is another goal for immune reconstitution in PWH on suppressive ART (Lu et al., 2015). In this context, previous studies have reported that only one-third of PWH on long-term ART achieve a restoration of the CD4^+^/CD8^+^ ratio, its normalization being slow (Caby, 2017; Han et al., 2018).

Metabolomics is based on studying and analyzing metabolites and metabolic pathways involved in a specific process. Serum and plasma derived from PWH revealed altered metabolites involved in lipid and mitochondrial pathways as well as fatty acids and organic acids (Cassol et al., 2013). The ability of ART to rectify HIV-induced metabolic dysregulation is unclear, and a robust characterization of the metabolic alterations experienced is needed to determine the effect of ART on these pathways. In this regards, Peltenburg et al. found increased lipid metabolites in PWH after 12 months of ART (Peltenburg et al., 2018). Regarding younger cohorts of PWH, significant changes in the levels of several metabolites were found between HIV untreated patients, HIV patients on ART, and healthy controls (Munshi et al., 2013). In PWH on long-term suppressive ART, Babu et al. (2019) reported plasma metabolomic abnormalities related to amino acids and energy metabolism, urea, and tricarboxylic acid cycle compared to healthy controls. Their findings also showed alterations in the lipid complex, which could be markers of inflammation, oxidative stress, and immune cell function. Gelpi et al. (2018) have also reported an independent association between HIV infection and hypertension, hypertriglyceridemia, and abdominal obesity. Nevertheless, they included PWH with a CD4^+^/CD8^+^ ratio <1, which could play a significant role in the differences. However, to our knowledge, no previous studies have evaluated metabolomic dysregulation in PWH on long-term ART with CD4^+^/CD8^+^ ≥1. In this population subgroup, these studies must assess the risk of inflammaging, immunosenescence, and age-related comorbidities.

This study aimed to determine whether the plasma metabolomic profile of PWH on long-term suppressive ART and immunologically recovered approximates the normality by comparison with healthy controls with similar age and gender.

## Methods

### Study subjects

We carried out a cross-sectional study in PWH (n=17) on long-term suppressive ART and significant immunological recovery in two Hospitals in Madrid (Hospital Universitario “La Paz” and Hospital General Universitario “Gregorio Marañón”). The selection criteria of PWH were: i) ART with HIV viral load <50 copies/ml and CD4^+^ T-cell counts ≥500 cells/mm^3^ during more than one year before blood extraction, and ii) CD4^+^/CD8^+^ ratio recovery to normal levels (≥1) at time of blood extraction. Patients with active hepatitis B virus (HBV) or hepatitis C virus (HCV) coinfections were excluded.

To evaluate normal plasma metabolite levels, we also selected a group of age- and gender-matched healthy controls (HC-group, n=19) that were negative for HIV, HBV, and HCV.

The study was approved by the Research Ethics Committee of the Institute of Health Carlos III (CEI PI 23_2011, CEI PI 41_2020-v2) and was carried out according to the Declaration of Helsinki. Before registration, all participants signed written consent.

### Clinical data and samples

Participant characteristics were collected from medical records. Peripheral blood samples were collected in EDTA tubes, and plasma samples were separated by centrifugation and stored at -80°C in the Spanish HIV HGM Biobank until use.

### Non-targeted metabolomics

The list of reagents and standards, metabolite extraction, and sample preparation are available in **Appendix A**. Metabolomic analysis was performed by two complementary analytical platforms: gas chromatography–mass spectrometry (GC-MS) system (Agilent Technologies 7890A) and liquid chromatography–mass spectrometry (LC-MS) (LC: 1290 infinity II Agilent, MS: Agilent 6550 iFunnel). Detailed methods can be found in **Appendix A**.

In GC-MS, the deconvolution and identification were performed using MassHunter Quantitative Unknowns Analysis (B.07.00, Agilent), alignment with MassProfiler Professional software (version 13.0, Agilent), and peak integration using MassHunter Quantitative Analysis (version B.07.00, Agilent). In LC-MS, the Molecular Feature Extraction and the Recursive Feature Extraction algorithms in the MassHunter Profinder software (B.08.00, Agilent) were used for the deconvolution and alignment of the raw data. After data reprocessing, the metabolic features were filtered (full description in **Supplementary File - Appendix A**).

### Multiplex immunoassays and ELISA

ProcartaPlexTM multiplex immunoassay (Bender MedSystems GmbH, Vienna, Austria) was used to measure several plasma biomarkers according to the manufacturer’s specifications using a Luminex 200TM analyzer (Luminex Corporation, Austin, TX, United States). The plasma biomarkers measured by ELISA multiplex were anti-inflammatory/suppressor markers – interleukin 10 (IL-10), transforming growth factor-beta 1 (TGF-β1), IL-1 receptor antagonist (IL-1RA) and IL-4 –, pro-inflammatory chemokine markers – human interferon-inducible protein 10 (IP-10), monocyte chemoattractant protein-1 (MCP-1)] and IL-8 –, pro-inflammatory cytokine markers – IL-1β, IL- 18, IL-6, tumor necrosis factor-alpha (TNF-α), interferon-gamma (IFN-γ), IL-12p70, IL-2 and IL-17A –, endothelial dysfunction markers – soluble vascular cell adhesion molecule-1 (sVCAM-1), soluble intercellular adhesion molecule-1 (sICAM-1) and soluble tumor necrosis factor receptor-1 (sTNFR-1) –, and coagulopathy markers – D-Dimer and plasminogen activator inhibitor-1 (PAI-1)–.

A Commercial ELISA was used to measure bacterial translocation markers – sCD14 and fatty acid-binding protein 2 (FABP2) (Raybiotech, Georgia, USA) and lipopolysaccharide-binding protein (LBP) (R&D Systems, Minneapolis, USA) – and the anti-transforming growth factor beta 1 (TGF-β1; Bender MedSystems GmbH, Vienna, Austria) as the multiplex immunoassay was not available. The lipopolysaccharide (LPS; Hycult Biotech, Uden, The Netherlands) was evaluated by a Limulus amebocyte lysate (LAL) chromogenic endpoint ELISA.

### Statistical analysis

For the group description, variables were expressed as median [25th; 75th percentile] for continuous and as absolute numbers [percentage] for categorical data. The Mann–Whitney U and Chi-square tests were used to analyze continuous and categorical variables, respectively.

For the metabolomics analysis, variables from GC-MS and LC-MS were log-transformed (log10) and auto-scaled to make individual features more comparable. Next, we performed an unsupervised analysis by principal component analysis (PCA) and a supervised analysis by partial least squares discriminant analysis (PLS-DA) for features detected in GC-MS and LC-MS [positive and negative electrospray ionization (ESI)]. The optimal number of PLS-DA components was determined with the leave-one-out cross-validation (LOOCV) method, using R^2^ and Q^2^ values as performance measures. Permutation was carried out by separation distance (B/W) with a permutation number of 1000 to confirm the model’s validity.

Generalized Linear Models (GLM) with gamma distribution (log-link) were used to independently analyze the differences between the study groups for each metabolite. This test provides the arithmetic mean ratio (AMR) and its significance level (p-value), which was corrected for multiple testing using the False Discovery Rate (FDR) with Benjamini and Hochberg procedure (q-value). Additionally, GLM models were adjusted by baseline characteristics (age, gender, and body mass index) previously selected by a stepwise method by the Akaike information criterion (AIC) (forward, p<0.05; q-value<0.20).

Correlation between significant metabolites and plasma biomarkers was performed using the Spearman correlation test. Those suitable correlations (r>0.5 or r<-0.5) and a significance value (p<0.05; q-value<0.20) were considered relevant.

The statistical analysis was done with MetaboAnalyst 4.0 software (http://www.metaboanalyst.ca/) and R statistical package version v3.5.1 (R Foundation for Statistical Computing, Vienna, Austria).

### Metabolite identification

The significant metabolites (q-value<0.2) were identified. In GC-MS, the identification was made based on FiehnLib (Kind et al., 2009) and NIST 14 libraries. In LC-MS, the list of accurate masses was searched using the CEU Mass Mediator search tool (http://ceumass.eps.uspceu.es/; error ± 5 ppm) to obtain tentative identifications. Each of them were manually curated based on their MS adducts (Salek et al., 2013; Godzien et al., 2016). In the cases that it was applicable, the elution order was also considered to discard spurious identifications. Eventually, the biological role of each compound was evaluated, and unrelated identifications such as pesticides, drugs, or not possible chemical structures were excluded. The metabolites are reported in agreement with the criteria of the Metabolomics Standards Initiative (10.1007/s11306-007-0070-6) with a confidence level grade 2 (putatively annotated compounds), which certitude is increased after manual curation of the final list.

## Results

### Patient characteristics

The epidemiological and clinical data of participants are shown in **Table 1**. In brief, the median age of PWH was 57 years, 64.7% were males, 47.1% had prior AIDS diagnosis, and the median time on ART was 10.7 years. Although all PWH had a CD4^+^/CD8^+^ ratio ≥1, healthy controls had significantly higher values (*p*-value<0.001).

**Table 1:** Clinical and epidemiological characteristics of PLW on long-term suppressive ART and healthy controls.

Characteristic	Healthy control	PWH	*p*-value
**No.**	19	17	
**Age (years)**	56 (51.5; 58.5)	57 (55.0; 58.0)	0.656
**Gender (male)**	10 (52.6%)	11 (64.7%)	0.693
**BMI (kg/m^2^)**	24.9 (23.8; 27.1)	25.6 (22.8; 27.9)	0.634
**Comorbidities**			
**Obesity (BMI>30)**	2 (10.5%)	3 (17.6%)	0.889
**Arterial hypertension**	2 (10.5%)	1 (5.9%)	0.999
**Diabetes**	0 (0.0%)	1 (5.9%)	0.999
**Risk group of HIV infection**			
**Heterosexual**	–	8 (47%)	–
**Homosexual**	–	9 (53%)	–
**Time of HIV infection (years)**	–	15.1 (10.9; 21.4)	–
**Antiretroviral therapy**			
**PI-based**	–	5 (29.4%)	–
**2NRTI+II-based**	–	1 (5.9%)	–
**2NRTI+NNRTI-based**	–	9 (52.9%)	–
**Others**	–	2 (11.8%)	–
**Antidepressant treatment**	–	2 (11.8%)	–
**Antihypertensive treatment**	–	3 (17.6%)	–
**Time on cART (years)**	–	10.7 (6.9; 16.3)	–
**Lymphocytes counts**			
**CD4^+^ T-cells (%)**	45.6 (42.9; 48.3)	37.7 (34.9; 43.3)	**0.003**
**CD8^+^ T-cells (%)**	15.7 (12.5; 21.1)	23.5 (20.4; 28.6)	**0.002**
**CD4^+^/CD8^+^**	2.7 (2.2; 3.6)	1.6 (1.2; 2.1)	**<0.001**
**CD4^+^/CD8^+^ ≥1**	19 (100%)	17 (100%)	0.999
**CD4^+^ T-cells/mm^3^**	–	977 (804; 1062)	–
**CD4^+^ ≥500 cells/mm^3^**	–	17 (100%)	–
**HIV markers**			
**Prior AIDS diagnosis**	–	8 (47.1%)	–
**Nadir CD4^+^ T-cells/mm^3^**	–	309 (64; 402)	–
**Nadir CD4^+^ ≤200 T-cells/mm^3^**	–	7 (41.2%)	–
**uVL (HIV-RNA <50 copies/mL)**	–	17 (100%)	–

**Statistics**: Values are expressed as absolute number (percentage) and median (interquartile range). P-values were calculated by Chi-square tests, Fisher's Exact Test or Mann-Whitney tests. Statistically significant differences are shown in bold.

**Abbreviations**: BMI, body mass index; HIV, human immunodeficiency virus; HIV-RNA, HIV plasma viral load; AIDS, acquired immune deficiency syndrome; NNRTI, non-nucleoside analogue HIV reverse transcriptase inhibitor; NRTI, nucleoside analogue HIV reverse transcriptase inhibitor; PI, protease inhibitor; II, integrase inhibitor; PWH, people with HIV; uVL, undetectable HIV viral load (<50 copies/mL).

### Reliability analysis

PCA indicated that quality control (QC) samples were tightly clustered together in the center of the plot, thus validating the signal stability and technical reproducibility (**Supplementary Figure 1**).

### Metabolite association analysis

**Figure 1:**
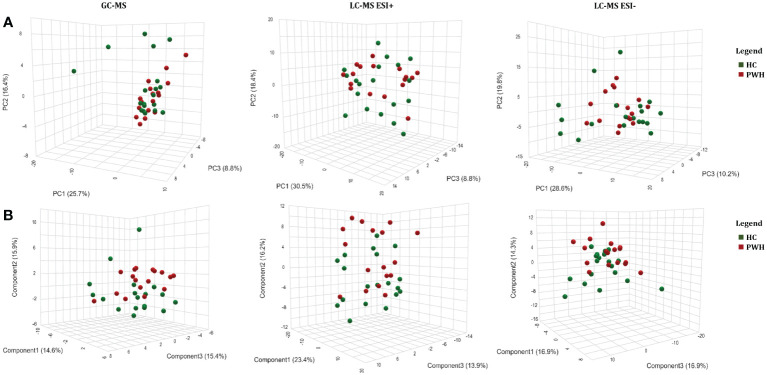
Multivariate metabolomic analysis in people with HIV (PWH) on long-term suppressive ART compared to healthy controls. **(A)** Principal component analysis (PCA) plot; **(B)** partial least squares - discriminant analysis (PLS-DA). PWH, people with human immunodeficiency virus; HC, healthy controls; GC-MS, gas chromatography–mass spectrometry; LC-MS, and liquid chromatography–mass spectrometry; ESI, electrospray ionization; PC, principal component.

PCA showed similarity between the sample groups for all the platforms used (**Figure 1A**). PLS-DA was performed for features detected in GC-MS (R^2^ = 0.377 and Q^2^ = 0.002; one component), LC-MS ESI+ (R^2^ = 0.658 and Q^2^=-0.076; three components), and LC-MS ESI- (R^2^ = 0.884 and Q^2^ = 0.232; five components) (**Figure 1B**). However, PLS-DA could not be validated by permutation for any of the platforms: GC-MS (p=0.323), LC-MS ESI+ (p=0.221), and LC-MS ESI- (p=0.548) (**Supplementary Figure 2**). Therefore, PCA and PLS-DA showed no significant differences between the two study groups. Similarly, differences between types of ART (PI-based, 2NRTI+NNRTI-based, and others) (**Supplementary Figure 3**) and nadir CD4+ levels (<200 cells/mm^3^ and ≥200 cells/mm^3^) (**Supplementary Figure 4**) in PWH group were not found.

GLM analysis adjusted by the most relevant covariates showed 63 significant features (p<0.05), of which 14 had a q<0.20 after correcting by FDR (**Table 2**). Of these, identification data were only obtained for three metabolites. Briefly, while lysophosphatidylcholine [LysoPC (22:6)] and lysophosphatidylethanolamine [LysoPE (22:6)] showed reduced levels [aAMR=0.59 (p=0.005; q=0.148) and aAMR=0.68 (p=0.001; q=0.050), respectively], an oxidized lipid not fully identified had increased levels [aAMR=1.11 (p=0.004; q=0.136)] among PWH compared to healthy controls (**Supplementary Figure 5**). The possible tentative identifications for this oxidized lipid were: 12-hydroperoxy-octadecatrienoic acid (HpOTrE), 13-HpOTrE, 13S-HpOTrE, 15,16-epoxy-octadecadienoic acid (EpODE), 16-HpOTrE, 7,8-dihydroperoxy-octadecatrienoic acid (DiHOTrE), 9H-12(13)-EpODE, 9-HpOTrE, or 9S-HpOTrE (HpOTrE/DiHOTrE/EpODE).

**Table 2:** Association of individual metabolites with HIV infection among PWH on long-term ART and immunologically recovered compared to healthy controls.

Feature	Technology	Mass	RT (min)	aAMR	IC2.5	IC97.5	p	q	Identification
Oleic acid	GC-MS	339	20.43	0.65	0.45	0.93	**0.024**	0.310	Oleic acid
Palmitic acid	GC-MS	313	18.84	0.71	0.53	0.96	**0.032**	0.310	Palmitic acid
Palmitoleic acid	GC-MS	311	18.65	0.60	0.38	0.95	**0.038**	0.310	Palmitoleic acid
p-Cresol	GC-MS	165	8.19	0.64	0.43	0.96	**0.039**	0.310	p-Cresol
Threonic acid	GC-MS	292	13.55	0.67	0.50	0.89	**0.010**	0.271	Threonic acid
Threonine	GC-MS	218	11.35	0.82	0.68	0.99	**0.046**	0.316	Threonine
Unknown_24.7	GC-MS	415	24.70	0.50	0.30	0.83	**0.012**	0.271	
Unknown_7.04	GC-MS	89	7.04	0.50	0.26	0.95	**0.040**	0.310	
Unknown_7.18	GC-MS	89	7.18	0.49	0.25	0.94	**0.039**	0.310	
Unknown_8.108	GC-MS	117	8.11	0.62	0.45	0.85	**0.006**	0.271	
414.2041/0.22399998	LC-MS ESI+	414.2041	0.22	1.18	1.03	1.35	**0.024**	0.270	
352.2021/0.25400043	LC-MS ESI+	352.2021	0.25	2.39	1.34	4.27	**0.006**	**0.148**	Unknown
416.2075/0.22399998	LC-MS ESI+	416.2075	0.22	1.27	1.07	1.51	**0.010**	**0.194**	Unknown
211.1932/6.314987	LC-MS ESI+	211.1932	6.31	1.12	1.01	1.24	**0.046**	0.307	
519.3325/5.4370084	LC-MS ESI+	519.3325	5.44	0.73	0.58	0.94	**0.019**	0.270	
267.2559/6.318981	LC-MS ESI+	267.2559	6.32	1.14	1.02	1.28	**0.033**	0.289	
297.268/0.2760002	LC-MS ESI+	297.268	0.28	1.14	1.03	1.26	**0.015**	0.240	
103.0999/5.572011	LC-MS ESI+	103.0999	5.57	0.76	0.60	0.95	**0.023**	0.270	
519.333/5.573987	LC-MS ESI+	519.333	5.57	0.73	0.55	0.97	**0.040**	0.295	
442.1838/0.26800057	LC-MS ESI+	442.1838	0.27	1.23	1.06	1.44	**0.012**	0.220	
307.2872/6.5200105	LC-MS ESI+	307.2872	6.52	1.19	1.03	1.37	**0.025**	0.270	
567.3322/5.5489993	LC-MS ESI+	567.3322	5.55	0.59	0.42	0.83	**0.005**	**0.148**	LysoPC(22:6)
326.2033/0.25500023	LC-MS ESI+	326.2033	0.26	1.13	1.04	1.24	**0.008**	**0.188**	Unknown
269.2718/6.687009	LC-MS ESI+	269.2718	6.69	1.17	1.02	1.35	**0.030**	0.285	
295.287/6.8239846	LC-MS ESI+	295.287	6.82	1.16	1.02	1.33	**0.028**	0.281	
796.155/9.532984	LC-MS ESI+	796.155	9.53	1.43	1.02	2.02	**0.045**	0.307	
729.2363/8.525017	LC-MS ESI+	729.2363	8.53	1.34	1.03	1.74	**0.038**	0.295	
798.1522/9.532984	LC-MS ESI+	798.1522	9.53	1.49	1.05	2.13	**0.034**	0.289	
806.2534/9.532984	LC-MS ESI+	806.2534	9.53	1.40	1.04	1.88	**0.032**	0.289	
117.0785/11.895997	LC-MS ESI+	117.0785	11.90	1.11	1.05	1.16	**<0.001**	**0.027**	Unknown
354.0627/10.401996	LC-MS ESI+	354.0627	10.40	1.33	1.02	1.72	**0.042**	0.300	
814.2073/10.39901	LC-MS ESI+	814.2073	10.40	1.43	1.06	1.93	**0.024**	0.270	
309.3033/7.241983	LC-MS ESI+	309.3033	7.24	1.18	1.01	1.36	**0.040**	0.295	
320.2461/0.27700037	LC-MS ESI+	320.2461	0.28	1.34	1.07	1.68	**0.015**	0.240	
283.2871/7.157991	LC-MS ESI+	283.2871	7.16	1.18	1.02	1.36	**0.032**	0.289	
946.1904/11.094979	LC-MS ESI+	946.1904	11.09	1.57	1.04	2.38	**0.040**	0.295	
944.1871/11.094977	LC-MS ESI+	944.1871	11.09	1.37	1.05	1.77	**0.024**	0.270	
281.2718/7.0589924	LC-MS ESI+	281.2718	7.06	1.21	1.01	1.46	**0.045**	0.307	
360.2254/0.26599964	LC-MS ESI+	360.2254	0.27	1.08	1.02	1.14	**0.010**	**0.194**	Unknown
103.0996/5.4420066	LC-MS ESI+	103.0996	5.44	0.73	0.61	0.89	**0.003**	**0.135**	Unknown
140.1059/11.895014	LC-MS ESI+	140.1059	11.90	1.14	1.05	1.24	**0.006**	**0.148**	Unknown
662.4444/11.645997	LC-MS ESI+	662.4444	11.65	1.13	1.01	1.27	**0.049**	0.307	
404.2528/0.28799918	LC-MS ESI+	404.2528	0.29	1.25	1.04	1.50	**0.021**	0.270	
877.2731/10.406988	LC-MS ESI+	877.2731	10.41	1.50	1.24	1.82	**<0.001**	**0.027**	Unknown
525.2854/5.403004	LC-MS ESI+	525.2854	5.40	0.68	0.55	0.84	**0.001**	**0.050**	LysoPE(22:6)
879.2761/10.407983	LC-MS ESI+	879.2761	10.41	1.60	1.26	2.02	**<0.001**	**0.027**	Unknown
879.2739/10.407982	LC-MS ESI+	879.2739	10.41	1.55	1.29	1.86	**<0.001**	**<0.001**	Unknown
870.1751/10.402992	LC-MS ESI+	870.1751	10.4	1.36	1.05	1.77	**0.027**	0.280	
340.2399/7.2280173	LC-MS ESI-	340.2399	7.23	0.23	0.06	0.92	**0.045**	0.708	
177.0801/0.25799963	LC-MS ESI-	177.0801	0.26	0.41	0.20	0.85	**0.022**	0.636	
188.0143/0.24000052	LC-MS ESI-	188.0143	0.24	0.50	0.30	0.82	**0.009**	0.397	
108.0572/0.24200036	LC-MS ESI-	108.0572	0.24	0.55	0.36	0.83	**0.008**	0.397	
886.5581/11.944002	LC-MS ESI-	886.5581	11.94	2.83	1.17	6.86	**0.027**	0.636	
251.1548/6.224987	LC-MS ESI-	251.1548	6.22	0.23	0.06	0.84	**0.033**	0.636	
404.2717/7.6960144	LC-MS ESI-	404.2717	7.70	0.23	0.09	0.61	**0.006**	0.397	
889.5749/11.930998	LC-MS ESI-	889.5749	11.93	2.22	1.04	4.74	**0.047**	0.708	
499.9411/0.257	LC-MS ESI-	499.9411	0.26	0.50	0.36	0.69	**<0.001**	**0.045**	Unknown
884.5422/11.938984	LC-MS ESI-	884.5422	11.94	2.55	1.11	5.81	**0.033**	0.636	
506.3392/8.925018	LC-MS ESI-	506.3392	8.93	0.27	0.09	0.88	**0.036**	0.636	
240.0729/7.214998	LC-MS ESI-	240.0729	7.21	0.43	0.23	0.82	**0.016**	0.545	
300.2091/6.3210077	LC-MS ESI-	300.2091	6.32	0.18	0.06	0.55	**0.005**	0.397	
713.4483/6.718995	LC-MS ESI-	713.4483	6.72	1.61	1.05	2.47	**0.036**	0.636	
310.2146/0.25799963	LC-MS ESI-	310.2146	0.26	1.11	1.04	1.18	**0.004**	**0.136**	HpOTrE/DiHOTrE/ EpODE *

**Statistics:** Generalized Linear Models (GLM) with a gamma distribution (log-link) (dependent variable: plasma metabolites; independent variable: HIV-infection), adjusted by epidemiological characteristics (age, gender and body mass index). P-values were adjusted by FDR correction for multiple comparisons (Benjamini and Hochberg). Statistically significant differences are shown in bold.

**Abbreviations**: RT, retention time; aAMR, adjusted ratio of the arithmetic means; CI, confidence interval; p-value, level of significance; q-value, adjusted p-value by FDR correction; GC-MS, gas chromatography–mass spectrometry; LC-MS ESI+, liquid chromatography–mass spectrometry, positive electrospray ionization; LC-MS ESI+, liquid chromatography–mass spectrometry, negative electrospray ionization; LysoPC, lysophosphocoline; LysoPE, lysophosphatidylethanolamine; HpOTrE, hydroperoxy-octadecatrienoic acid; DiHOTrE, dihydroperoxy-octadecatrienoic acid; EpODE, epoxy-octadecadienoic acid.

* 12-HpOTrE / 13-HpOTrE / 13S-HpOTrE / 15,16-EpODE / 16-HpOTrE / 7,8-DiHOTrE / 9H-12(13)-EpODE / 9-HpOTrE / 9S-HpOTrE.

### Correlation analysis between metabolites and plasma biomarkers

**Figure 2:**
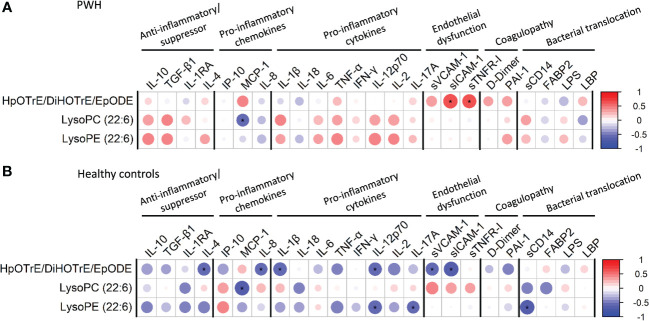
Spearman correlation plot between significant metabolites and plasma cytokines/chemokines: **(A)** people with HIV (PWH); **(B)** healthy controls. The size of the circles is proportional to the strength of the correlation, and the color represents the direction (color legends are shown on the right), where large dark blue represents a strong negative correlation, and a large dark red circle represents a strong positive correlation. Metabolites are on the vertical axis, and cytokines/chemokines are on the horizontal axis. Those correlations with rho>0.5 o rho<-0.5, p-value<0.05, and q-value<0.2 are shown with an asterisk. IL, interleukin; TGF-β1, transforming growth factor beta 1; IL-1RA, IL-1 receptor antagonist; IP-10, human interferon-inducible protein 10; MCP-1, monocyte chemoattractant protein-1; TNF-α, tumor necrosis factor alpha; IFN-γ, interferon gamma; sVCAM-1, soluble vascular cell adhesion molecule-1; sICAM-1, soluble intercellular adhesion molecule-1; sTNFR-1, soluble tumour necrosis factor receptor-1; PAI-1, plasminogen activator inhibitor-1; FABP2, fatty acid-binding protein 2; LPS, lipopolysaccharide; LBP, lipopolysaccharide binding protein.

The plasma biomarkers concentrations in both HC and PWH groups are shown in **Supplementary Table 1**. The correlations between significant identified metabolites and plasma biomarkers are shown in **Figure 2** (full description in **Supplementary Tables 2**, **3**). Several correlations were found significant, even after FDR correction (r>0.5 or r<-0.5; p<0.05; q-value<0.20). LysoPC (22:6) was negatively correlated with MCP-1 in PWH and the HC-group (p=0.037 and p=0.002, respectively). Besides, while no significant correlations were found for LysoPE (22:6) in PWH, negative correlations were found between LysoPE (22:6) and IL-12p70 (p=0.012), IL-17A (p=0.040), and sCD14 (p=0.002) in the HC-group. HpOTrE/DiHOTrE/EpODE was also positively correlated with sICAM-1 (p=0.004) and sTNFR-I (p=0.009) in PWH. However, we found negative correlations between HpOTrE/DiHOTrE/EpODE and sICAM-1 (p=0.001), sVCAM-1 (p=0.016), IL-12p70 (p=0.018), IL-1β (p=0.011), IL-8 (p=0.027), IL-4 (p=0.021) in the HC-group.

## Discussion

We found little differences in the metabolic profile between PWH with immunological recovery after long-term suppressive ART and healthy controls.

PCA and PLS-DA multivariate analysis showed no relevant results for any platforms used. Previous metabolomic studies have shown a clear separation between PWH and HC groups (Hewer et al., 2006; Herbert et al., 2023), even for PWH on long-term successful antiretroviral therapy (Babu et al., 2019), in which alterations in amino-acid levels, energetics, and lipids have been found. However, PWH did not achieve a CD4/CD8 ratio >1 in most studies, which could contribute to the differences observed in these studies in contrast to ours. Besides, several articles have described a metabolomic signature associated with immunological CD4+ T-cell recovery after long-term of antiretroviral therapy. However, it has been also studied in PWH whose immunological recovery did not exceed the CD4 T-cell count of 500 (Rodriguez-Gallego et al., 2018; Nystrom et al., 2021; Qian et al., 2021), which limits comparisons with our study. In this sense, different overlapping metabolic profiles between PWH and HC has been found probably due to different disease stage of individuals (Williams et al., 2012), which indicates that the characteristics of patients and their level of immunological recovery are crucial to interpret the findings. Thus, further studies including PWH with longer periods of successful ART and improved immune reconstitution (CD4^+^/CD8^+^ ≥1) would be needed to corroborate the near-normal metabolomic profile found in our study.

However, although, to our knowledge, no previous metabolomic studies have been performed in PWH on long-term ART with CD4^+^/CD8^+^ ≥1compared to healthy controls, the finding of an almost normalization of the metabolic profile in this subgroup of PWH is concordant with previous studies, in which a normalization of different immune-related molecular markers has been described. In this same cohort, Brochado-Kith et al. showed that peripheral blood mononuclear cells gene expression and peripheral blood biomarkers in PWH, with normalized CD4^+^/CD8^+^ ratio, had a similar profile compared to healthy controls (BroChado-Kith et al., 2020). Serrano-Villar et al. found that PWH on ART with a normalized CD4^+^/CD8^+^ ratio demonstrated traits of a nearly healthy immune system (Serrano-Villar et al., 2014). Sperk et al. showed that some pro-inflammatory cytokines and chemokines return to healthy levels in PWH with nearly twenty years of ART (Sperk et al., 2018).

Additionally, the GLM analysis of each metabolite showed scarce differences between groups. Decreased levels of lysoPCs (22:6) and lysoPE (22:6) were found in PWH on long-term ART compared to healthy controls. Although these specific metabolites have not been described in previous studies, an altered level of phosphatidylcholine and phosphatidylethanolamine has been found comparing PWH on long-term successful antiretroviral therapy and HC groups (Babu et al., 2019). Likewise, Lu et al. described that glycerophospholipid metabolism was one of the pathways with highest impact (Lu et al., 2023), which is in line with our findings. Additionally, lower level of these lysophospholipids has been also associated to other human diseases, such as metabolic, cardiovascular, and neurodegenerative disorders, all non-AIDS-defining events (NADEs) among PWH (Law et al., 2019). Likewise, lower lysoPCs (22:6) and lysoPE (22:6) levels have also been associated with more advanced cirrhosis stages among HIV/HCV-coinfected patients (Salguero et al., 2020). In addition, decreased concentrations of different LysoPCs species have been associated with the risk of obesity (Barber et al., 2012), linked to inflammaging, insulin resistance, metabolic syndrome, and non-alcoholic fatty liver disease, among others. Regarding ART, several diabetes-associated lipid species are perturbed in ART-treated PWH, as ART disrupts lipid metabolism (Brown and Glesby, 2011).

The metabolites whose levels differed between groups have been associated with inflammaging and immune activation (Lee et al., 2016; Toledo et al., 2017; Law et al., 2019), which can lead to premature aging and NADEs (Zicari et al., 2019). In this setting, we analyzed the correlation of these metabolites with several plasma biomarkers and found a significant negative correlation between LysoPC (22:6) and the pro-inflammatory chemokine MCP-1 in PWH, supporting the inflammaging and immune activation state in these patients. No significant correlations were found for LysoPE (22:6) in PWH. Regarding the oxidized lipid, significant positive correlations were found with sICAM-1 and sTNFR-1 in PWH, both being endothelial dysfunction markers and associated with increased risk of cardiovascular disease, cancers, and atherosclerosis, among others (Gross et al., 2012; Zicari et al., 2019; Bui et al., 2020). Interestingly, phospholipid metabolism has been previously found to be altered in studies including younger PWH on long-term ART compared HC (mean age of 45 years in both groups) (Babu et al., 2019; Lu et al., 2023), which could indicate that similar molecular mechanisms may also occur in younger cohorts, although further studies are needed.

Although the introduction of ART has increased the life expectancy of PWH (Zicari et al., 2019), our study suggests it does not restore health *ad-integrum*. We observed that even patients with a normalized CD4^+^/CD8^+^ ratio presented alteration of specific metabolites that could be involved in the pathogenesis of different age-related comorbidities due to chronic immune activation, immunosenescence, and inflammaging.

Therefore, it is essential to carry out further studies that corroborate the role of metabolic changes during ART among PWH. Likewise, metabolomics can offer an alternative view of the inflammatory state of patients.

Some limitations should be considered for a correct interpretation of the data. Firstly, the sample size was limited, which could have restricted the statistical power to detect metabolic differences between groups. In addition, the modest sample size may also increase the false positive rate, but our positive findings were FDR-corrected, lending robustness to our results. Second, this study has a cross-sectional design, which may introduce some bias and limit the interpretation of our findings. Thirdly, clinical data related to the individual cognitive status were unavailable, which would be interesting as neurocognitive disorders have been shown to impact the lipidome. Finally, more studies would be needed in other patient cohorts, such as among PWH with different age ranges, to confirm whether similar results are found.

## Conclusions

In conclusion, our data suggests that PWH on long-term ART, with CD4+/CD8+ ratio ≥1, have a metabolomic profile that is almost normal compared to healthy controls. Nevertheless, residual metabolic alterations linked to inflammatory biomarkers persist, which could favor the development of age-related comorbidities among this population.

## Data availability statement

The original contributions presented in the study are included in the article/**Supplementary Material**. Further inquiries can be directed to the corresponding author.

## Ethics statement

The studies involving humans were approved by The study was approved by the Research Ethics Committee of the Institute of Health Carlos III (CEI PI 23_2011, CEI PI 41_2020-v2) and was carried out according to the Declaration of Helsinki. Before registration, all participants signed written consent. The studies were conducted in accordance with the local legislation and institutional requirements. The participants provided their written informed consent to participate in this study.

## Author contributions

AV-B: Data curation, Formal analysis, Investigation, Methodology, Visualization, Writing – original draft. RM-E: Data curation, Formal analysis, Investigation, Visualization, Writing – original draft. JB: Conceptualization, Investigation, Writing – review & editing. JG-G: Investigation, Writing – review & editing. OB-K: Formal analysis, Investigation, Writing – review & editing. DR: Investigation, Writing – review & editing. AF-R: Investigation, Writing – review & editing. LP-L: Investigation, Writing – review & editing. VH: Investigation, Writing – review & editing. CB: Investigation, Writing – review & editing. SR: Conceptualization, Funding acquisition, Project administration, Resources, Supervision, Validation, Visualization, Writing – review & editing. MAJ-S: Conceptualization, Funding acquisition, Project administration, Resources, Supervision, Validation, Visualization, Writing – review & editing.

## References

[B1] Babu H. Sperk M. Ambikan A. T. Rachel G. Viswanathan V. K. Tripathy S. P.. (2019). Plasma metabolic signature and abnormalities in HIV-infected individuals on long-term successful antiretroviral therapy. Metabolites 9 (10), 210. doi: 10.3390/metabo9100210 31574898 PMC6835959

[B2] Barber M. N. Risis S. Yang C. Meikle P. J. Staples M. Febbraio M. A.. (2012). Plasma lysophosphatidylcholine levels are reduced in obesity and type 2 diabetes. PloS One 7 (7), e41456. doi: 10.1371/journal.pone.0041456 22848500 PMC3405068

[B3] BroChado-Kith O. Martinez I. Berenguer J. Medrano L. M. Gonzalez-Garcia J. Garcia-Broncano P.. (2020). Near normalization of peripheral blood markers in HIV-infected patients on long-term suppressive antiretroviral therapy: a case-control study. AIDS 34 (13), 1891–1897. doi: 10.1097/QAD.0000000000002645 32796212

[B4] Brown T. T. Glesby M. J. (2011). Management of the metabolic effects of HIV and HIV drugs. Nat. Rev. Endocrinol. 8 (1), 11–21. doi: 10.1038/nrendo.2011.151 21931374 PMC3371609

[B5] Bui T. M. Wiesolek H. L. Sumagin R. (2020). ICAM-1: A master regulator of cellular responses in inflammation, injury resolution, and tumorigenesis. J. Leukoc. Biol. 108 (3), 787–799. doi: 10.1002/JLB.2MR0220-549R 32182390 PMC7977775

[B6] Caby F. (2017). CD4+/CD8+ ratio restoration in long-term treated HIV-1-infected individuals. AIDS 31 (12), 1685–1695. doi: 10.1097/QAD.0000000000001533 28700392

[B7] Cassol E. Misra V. Holman A. Kamat A. Morgello S. Gabuzda D. (2013). Plasma metabolomics identifies lipid abnormalities linked to markers of inflammation, microbial translocation, and hepatic function in HIV patients receiving protease inhibitors. BMC Infect. Dis. 13, 203. doi: 10.1186/1471-2334-13-203 23641933 PMC3655873

[B8] Chen J. Liu X. Qin S. Ruan G. Lu A. Zhang J.. (2022). A novel prognostic score including the CD4/CD8 for AIDS-related lymphoma. Front. Cell Infect. Microbiol. 12, 919446. doi: 10.3389/fcimb.2022.919446 35873145 PMC9299417

[B9] Gelpi M. Afzal S. Lundgren J. Ronit A. Roen A. Mocroft A.. (2018). Higher Risk of Abdominal Obesity, Elevated Low-Density Lipoprotein Cholesterol, and Hypertriglyceridemia, but not of Hypertension, in People Living With Human Immunodeficiency Virus (HIV): Results From the Copenhagen Comorbidity in HIV Infection Study. Clin. Infect. Dis. 67 (4), 579–586. doi: 10.1093/cid/ciy146 29471519

[B10] Godzien J. Ciborowski M. Armitage E. G. Jorge I. Camafeita E. Burillo E.. (2016). A single in-vial dual extraction strategy for the simultaneous lipidomics and proteomics analysis of HDL and LDL fractions. J. Proteome Res. 15 (6), 1762–1775. doi: 10.1021/acs.jproteome.5b00898 27117984

[B11] Gross M. D. Bielinski S. J. Suarez-Lopez J. R. Reiner A. P. Bailey K. Thyagarajan B.. (2012). Circulating soluble intercellular adhesion molecule 1 and subclinical atherosclerosis: the Coronary Artery Risk Development in Young Adults Study. Clin. Chem. 58 (2), 411–420. doi: 10.1373/clinchem.2011.168559 22179741 PMC3867124

[B12] Han W. M. Apornpong T. Kerr S. J. Hiransuthikul A. Gatechompol S. Do T.. (2018). CD4/CD8 ratio normalization rates and low ratio as prognostic marker for non-AIDS defining events among long-term virologically suppressed people living with HIV. AIDS Res. Ther. 15 (1), 13. doi: 10.1186/s12981-018-0200-4 30261902 PMC6158807

[B13] Herbert C. Luies L. Loots D. T. Williams A. A. (2023). The metabolic consequences of HIV/TB co-infection. BMC Infect. Dis. 23 (1), 536. doi: 10.1186/s12879-023-08505-4 37592227 PMC10436461

[B14] Hewer R. Vorster J. Steffens F. E. Meyer D. (2006). Applying biofluid 1H NMR-based metabonomic techniques to distinguish between HIV-1 positive/AIDS patients on antiretroviral treatment and HIV-1 negative individuals. J. Pharm. BioMed. Anal. 41 (4), 1442–1446. doi: 10.1016/j.jpba.2006.03.006 16621406

[B15] Kind T. Wohlgemuth G. Lee D. Y. Lu Y. Palazoglu M. Shahbaz S.. (2009). FiehnLib: mass spectral and retention index libraries for metabolomics based on quadrupole and time-of-flight gas chromatography/mass spectrometry. Anal. Chem. 81 (4), 10038–10048. doi: 10.1021/ac9019522 19928838 PMC2805091

[B16] Law S. H. Chan M. L. Marathe G. K. Parveen F. Chen C. H. Ke L. Y. (2019). An updated review of lysophosphatidylcholine metabolism in human diseases. Int. J. Mol. Sci. 20 (5), 1149. doi: 10.3390/ijms20051149 30845751 PMC6429061

[B17] Lee J. M. Lee H. Kang S. Park W. J. (2016). Fatty acid desaturases, polyunsaturated fatty acid regulation, and biotechnological advances. Nutrients 8 (1), 23. doi: 10.3390/nu8010023 26742061 PMC4728637

[B18] Lu L. Yang Y. Yang Z. Wu Y. Liu X. Li X.. (2023). Altered plasma metabolites and inflammatory networks in HIV-1 infected patients with different immunological responses after long-term antiretroviral therapy. Front. Immunol. 14, 1254155. doi: 10.3389/fimmu.2023.1254155 37828979 PMC10565217

[B19] Lu W. Mehraj V. Vyboh K. Cao W. Li T. Routy J. P. (2015). CD4:CD8 ratio as a frontier marker for clinical outcome, immune dysfunction and viral reservoir size in virologically suppressed HIV-positive patients. J. Int. AIDS Soc. 18 (1), 20052. doi: 10.7448/IAS.18.1.2005220052 26130226 PMC4486418

[B20] Munshi S. U. Rewari B. B. Bhavesh N. S. Jameel S. (2013). Nuclear magnetic resonance based profiling of biofluids reveals metabolic dysregulation in HIV-infected persons and those on anti-retroviral therapy. PloS One 8 (5), e64298. doi: 10.1371/journal.pone.0064298 23696880 PMC3655987

[B21] Nystrom S. Govender M. Yap S. H. Kamarulzaman A. Rajasuriar R. Larsson M. (2021). HIV-infected individuals on ART with impaired immune recovery have altered plasma metabolite profiles. Open Forum Infect. Dis. 8 (7), ofab288. doi: 10.1093/ofid/ofab288 34258318 PMC8271132

[B22] Peltenburg N. C. Schoeman J. C. Hou J. Mora F. Harms A. C. Lowe S. H.. (2018). Persistent metabolic changes in HIV-infected patients during the first year of combination antiretroviral therapy. Sci. Rep. 8 (1), 16947. doi: 10.1038/s41598-018-35271-0 30446683 PMC6240055

[B23] Qian S. Chen X. Wu T. Sun Y. Li X. Fu Y.. (2021). The accumulation of plasma acylcarnitines are associated with poor immune recovery in HIV-infected individuals. BMC Infect. Dis. 21 (1), 808. doi: 10.1186/s12879-021-06525-6 34384363 PMC8362229

[B24] Rodriguez-Gallego E. Gomez J. Pacheco Y. M. Peraire J. Vilades C. Beltran-Debon R.. (2018). A baseline metabolomic signature is associated with immunological CD4+ T-cell recovery after 36 months of antiretroviral therapy in HIV-infected patients. AIDS 32 (5), 565–573. doi: 10.1097/QAD.0000000000001730 29280761 PMC5844590

[B25] Salek R. M. Steinbeck C. Viant M. R. Goodacre R. Dunn W. B. (2013). The role of reporting standards for metabolite annotation and identification in metabolomic studies. Gigascience 2 (1), 13. doi: 10.1186/2047-217X-2-13 24131531 PMC3853013

[B26] Salguero S. Rojo D. Berenguer J. Gonzalez-Garcia J. Fernandez-Rodriguez A. BroChado-Kith O.. (2020). Plasma metabolomic fingerprint of advanced cirrhosis stages among HIV/HCV-coinfected and HCV-monoinfected patients. Liver Int. 40 (9), 2215–2227. doi: 10.1111/liv.14580 32593189

[B27] Serrano-Villar S. Sainz T. Lee S. A. Hunt P. W. Sinclair E. Shacklett B. L.. (2014). HIV-infected individuals with low CD4/CD8 ratio despite effective antiretroviral therapy exhibit altered T cell subsets, heightened CD8+ T cell activation, and increased risk of non-AIDS morbidity and mortality. PloS Pathog. 10 (5), e1004078. doi: 10.1371/journal.ppat.1004078 24831517 PMC4022662

[B28] Sperk M. Zhang W. Nowak P. Neogi U. (2018). Plasma soluble factor following two decades prolonged suppressive antiretroviral therapy in HIV-1-positive males: A cross-sectional study. Med. (Baltimore) 97 (5), e9759. doi: 10.1097/MD.0000000000009759 PMC5805434 29384862

[B29] Toledo E. Wang D. D. Ruiz-Canela M. Clish C. B. Razquin C. Zheng Y.. (2017). Plasma lipidomic profiles and cardiovascular events in a randomized intervention trial with the Mediterranean diet. Am. J. Clin. Nutr. 106 (4), 973–983. doi: 10.3945/ajcn.116.151159 28814398 PMC5611779

[B30] Williams A. Koekemoer G. Lindeque Z. Reinecke C. Meyer D. (2012). Qualitative serum organic acid profiles of HIV-infected individuals not on antiretroviral treatment. Metabolomics 8, 804–818. doi: 10.1007/s11306-011-0376-2

[B31] Zicari S. Sessa L. Cotugno N. Ruggiero A. Morrocchi E. Concato C.. (2019). Immune activation, inflammation, and non-AIDS co-morbidities in HIV-infected patients under long-term ART. Viruses 11 (3), 200. doi: 10.3390/v11030200 30818749 PMC6466530

