# Supplementary material for: Plasma metabolomic profile is near-normal in people with HIV on long-term suppressive antiretroviral therapy
Source: Front Cell Infect Microbiol. 2024 Mar 14;14:1340610. doi: 10.3389/fcimb.2024.1340610 (PMC10972849; doi:10.3389/fcimb.2024.1340610)
Supplement: Supplementary file 1 [file DataSheet_1.docx]

# Appendix A: Additional description of methods section

***Reagents and standards for metabolomics***

The following was used: acetonitrile (LC-MS grade, Sigma-Aldrich, Steinheim, Germany), formic acid (FA) (MS grade, Sigma-Aldrich, Steinheim, Germany), MilliQ® water (Millipore, Billerica, MA, USA), heptane (Sigma-Aldrich, Steinheim, Germany), methylterbutyleter (MTBE) (Sigma-Aldrich, Steinheim, Germany), pyridine (Sigma-Aldrich, Steinheim, Germany), O-methoxyamine hydrochloride (Sigma-Aldrich, Steinheim, Germany) and N,O-bis(trimethylsilyl) trifluoroacetamide (BSTFA) plus 1 % trimethylchlorosilane (TMCS) (Pierce Chemical Co, Rockford, IL, USA). Stearic acid methyl ester (C18:0 methyl ester) (Sigma-Aldrich, Steinheim, Germany) was used as an internal standard for GC-MS. For reference masses purine, hexakis(1H,1H,3H-tetrafluoropropoxy)phosphazine (HP), and ammonium trifluoroacetate (TFA(NH_4_)) from Agilent (API-TOF reference mass solution kit) were used in LC-MS. In GC-MS, a FAME mix (fatty acid methyl esters, i.e. caprylic acid, capric acid, lauric acid, tridecanoic acid, myristic acid, myristoleic acid, pentadecanoic acid, palmitic acid, palmitoleic acid, heptadecanoid acid, stearic acid, elaidic acid, oleic acid, linoleic acid, arachidic acid, cis-11-eicosenoic acid, linolenic acid, behenic acid and erucic acid) was purchased from Supelco (Bellefonte, PA, USA).

***Sample preparation for metabolomics analysis***

Firstly, plasma samples were inactivated for viruses by mixing methanol for ultra-performance liquid chromatography (UPLC) supergradient with plasma (3:1, v/v). Next, vortexing (15 s), maintenance in cold for 5 min, centrifugation (16000 g, 20 min, 4°C) and freezing at 80°C were performed before the shipment of samples to the Center for Metabolomics and Bioanalysis (San Pablo-CEU University, Spain). On the day of the analysis, for GC-MS 100 µL of the corresponding methanolic aliquots were evaporated to dryness using a Speedvac Concentrator, followed by the addition of 10 μL of O-methoxyamine hydrochloride (15 mg/mL) in pyridine for methoximation. After gently vortexing, the vials were incubated in darkness at room temperature for 16 hours. Then, 10 μL of BSTFA with 1 % TMCS (v/v) were added, and samples were vortexed for 5 min. Silylation was carried out for 1 h at 70°C, and finally, 100 μL of C18:0 methyl ester (10 mg/L in heptane) were added as an internal standard, and samples were mixed again by gently vortex. Six blank samples were prepared by the same procedure of extraction and derivatization. For LC-MS, 500 µL of MTBE were added to the samples to enhance the extraction of the lipophilic compounds. After gently vortexing (TissueLyser LT, 50 Hz, 10 min) and centrifugation (16000 g, 20 min, 4°C), the resulting supernatants were filtered through 0.22 µm nylon syringe filters and transferred into an analytical vial for their analysis.

Metabolomic analysis was performed by two complementary analytical platforms. On the one hand, it was used a GC system (Agilent Technologies 7890A). The derivatized samples were injected through a GC-Column DB5-MS with a pre-column (10 m J&W integrated with Agilent 122-5532G). On the other hand, it was used a LC system consisting of a degasser, a binary pump, and an autosampler (1290 infinity II, Agilent). The samples were applied to a reversed-phase column (Zorbax Extend C18 50 x 2.1 mm, 1.8 µm; Agilent). Data were collected in positive and negative electrospray ionization (ESI) modes in separate runs using Q-TOF (Agilent 6550 iFunnel).

Quality control (QCs) samples are required at the beginning of the sequence to stabilize the system and throughout the analytical runs at periodic intervals to monitor variations in signal across the time. For these reasons, individual QC samples were prepared independently for each analytical platform by pooling and mixing equal volumes of each corresponding sample. After gently vortex, the mixes were transferred to analytical vials. While individual QCs were used for GC, one QC was prepared and reinjected several times for LC.

***GC-EI-Q-MS fingerprinting*** (for using FiehnLib [1] and NIST 14 libraries)

GC system (Agilent Technologies 7890A) consisted of an autosampler (Agilent Technologies 7693) and an inert mass selective detector (MSD) with Quadrupole (Agilent Technologies 5975). Two μL of the derivatized sample were injected through a GC-Column DB5-MS (30 m length, 0.25 mm internal diameter, 0.25 μm film 95% dimethylpolysiloxane / 5% diphenylpolysiloxane) with a pre-column (10 m J&W integrated with Agilent 122-5532G). The flow rate of the helium carrier gas was set at 1 mL/min, and the injector temperature was 250°C. The split ratio was 1:10 flow into a Restek 20782 deactivated glass-wool split liner. The temperature gradient was programmed at 60°C (held for 1 min), with a ramping increase rate of 10 °C/min up to 325°C. Finally, it was cooled down for 10 min before the next injection. The total analysis time was 37.5 min. The detector transfer line, filament source, and quadrupole temperature were respectively set at 290°C, 230°C, and 150°C. The electron ionization (EI) source was placed at 70 eV. The mass spectrometer operated in scan mode over a mass range of *m/z* 50–600 at a rate of 2 spectra per second. The method was retention time locked at 19.663 minutes (elution time of the internal standard). The analytical run was set up starting with the injection of C18:0 methyl ester (10 mg/L in heptane) and FAME mix (0.1 mg/mL in CH_2_Cl_2_) followed by four blanks, five QCs, and then samples were analyzed in a randomized order, where other QCs were injected between blocks of ten samples until the end of the run that terminated with the injection of the four blanks. Normalization by the internal standard was carried out.

***LC-ESI-QTOF-MS fingerprinting***

The metabolic profile was achieved using a liquid chromatography system consisting of a degasser, a binary pump, and an autosampler (1290 infinity II, Agilent). Samples (0.5 µL) were applied to a reversed-phase column (Zorbax Extend C18 50 x 2.1 mm, 1.8 µm; Agilent), which was maintained at 60°C during the analysis. The system was operated at a flow rate of 0.6 mL/min with solvent A (H_2_O containing 0.1% FA) and solvent B (acetonitrile containing 0.1% FA). The gradient was 5% B (0–1 min), 5 to 80% B (1–7 min), 80 to 100% B (7–11.5 min), and 100 to 5% B (11.5–12 min). The system was finally held at 5% B for 3 min to re-equilibrate the system (15 min of total analysis time). Data were collected in positive and negative electrospray ionization (ESI) modes in separate runs using QTOF (Agilent 6550 iFunnel). The analyses were performed in both positive and negative ion modes in full-scan from *m/z* 50 to 1000. The capillary voltage was 3000 V, and the nozzle voltage was 1000 V with a scan rate of 1.0 spectrum per second. The gas temperature was 250°C, the drying gas flow was 12 L/min, the nebulizer was 52 psi, the sheath gas temperature 370°C, and the sheath gas flow 11 L/min. For positive mode, the MS-TOF parameters were as follows: fragmentor 175 V and octopole radio frequency voltage 750 V. For negative mode, the MS-TOF parameters included the following: fragmentor 250 V and octopole radio frequency voltage 750 V. During the analyses, two reference masses were used: 121.0509 (purine, detected *m/z* [C_5_H_4_N_4_+H]^+^) and 922.0098 (HP, detected *m/z* [C_18_H_18_O_6_N_3_P_3_F_24_+H]^+^) in positive mode and 112.9855 (TFA(NH_4_), detected *m/z* [C_2_O_2_F_3_(NH_4_)-H]^-^) and 966.0007 (HP+FA, detected *m/z* [C_18_H_18_O_6_N_3_P_3_F_24_+FA-H]^-^) in negative mode. The references were continuously infused into the system, enabling constant mass correction. Samples were analyzed in randomized runs, during which they were incubated in an autosampler at 4°C. The analytical runs for both polarities were set up starting with the analysis of ten QCs followed by the samples; a QC sample was injected between blocks of ten samples until the end of the run. A total of 13 blank samples were used. At the beginning, three blank samples were used to equilibrate the chromatographic system. Next, other 5 blank samples were injected at the beginning and at the end as analytical blanks. These blank samples were also compared to confirm the absence of carryover between samples.

***Quality assurance***

After data reprocessing, the metabolic features were subsequently filtered. For GC-MS, 82 metabolites were detected. After filtering for a relative standard deviation (RSD) <40 and presence in at least 60% of the samples in each experimental group, 68 metabolites were selected for statistical analysis. For LC-MS, a normalization over the analysis time (injection order) was carried [2]. The detected features were 347 and 335 for ESI+ and ESI-, respectively. Of them, 261 and 210 features fulfilled a value of RSD <40, of which 13 and 2 features had no presence in more than 60% of samples in each group and were discarded. Finally, 248 and 208 features were statistically analyzed for ESI+ and ESI-, respectively.

**REFERENCES**

1. Kind T, Wohlgemuth G, Lee dY, et al. FiehnLib: mass spectral and retention index libraries for metabolomics based on quadrupole and time-of-flight gas chromatography/mass spectrometry. Anal Chem **2009**; 81:10038-48.

2. Kuligowski J, Sanchez-Illana A, Sanjuan-Herraez D, Vento M, Quintas G. Intra-batch effect correction in liquid chromatography-mass spectrometry using quality control samples and support vector regression (QC-SVRC). Analyst **2015**; 140:7810-7.

**Supplementary Figure 1.** Principal component analysis (PCA) plot showing clustering of study samples and quality controls.


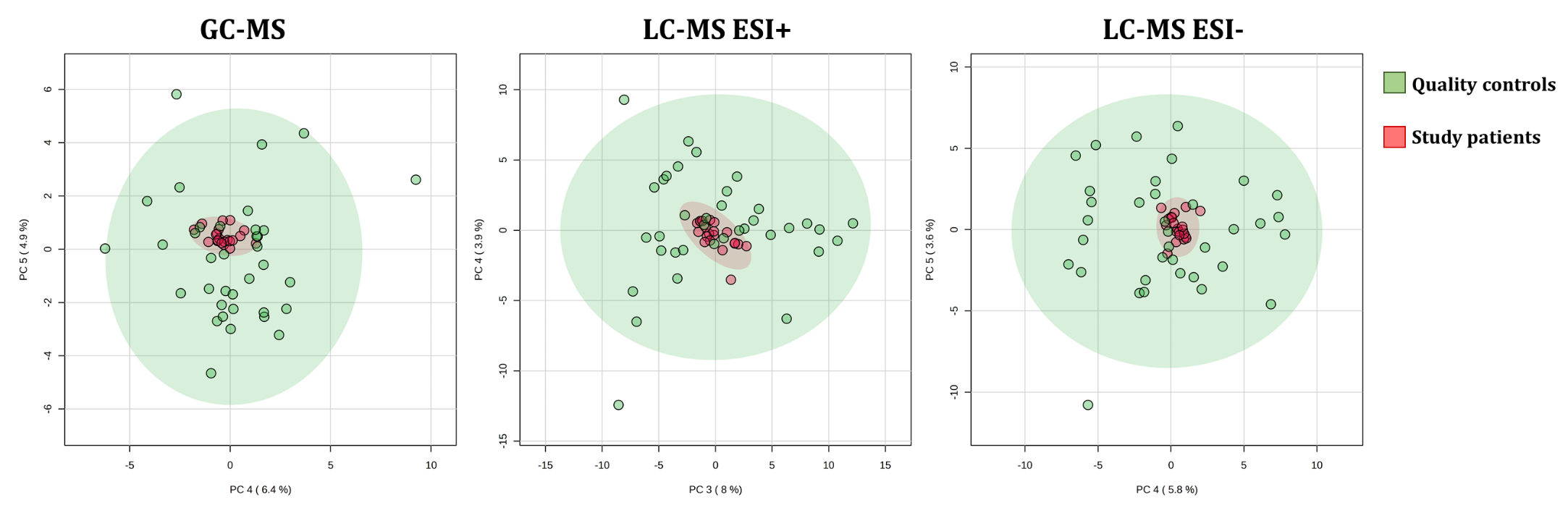


**Abbreviations:** GC-MS, gas chromatography–mass spectrometry; LC-MS, and liquid chromatography–mass spectrometry; ESI, electrospray ionization; PC, principal component.

**Supplementary Figure 2.** Permutation by separation distance (B/W) to confirm the validity of the PLS-DA models for both GC and LC data, with a permutation number of 1000.


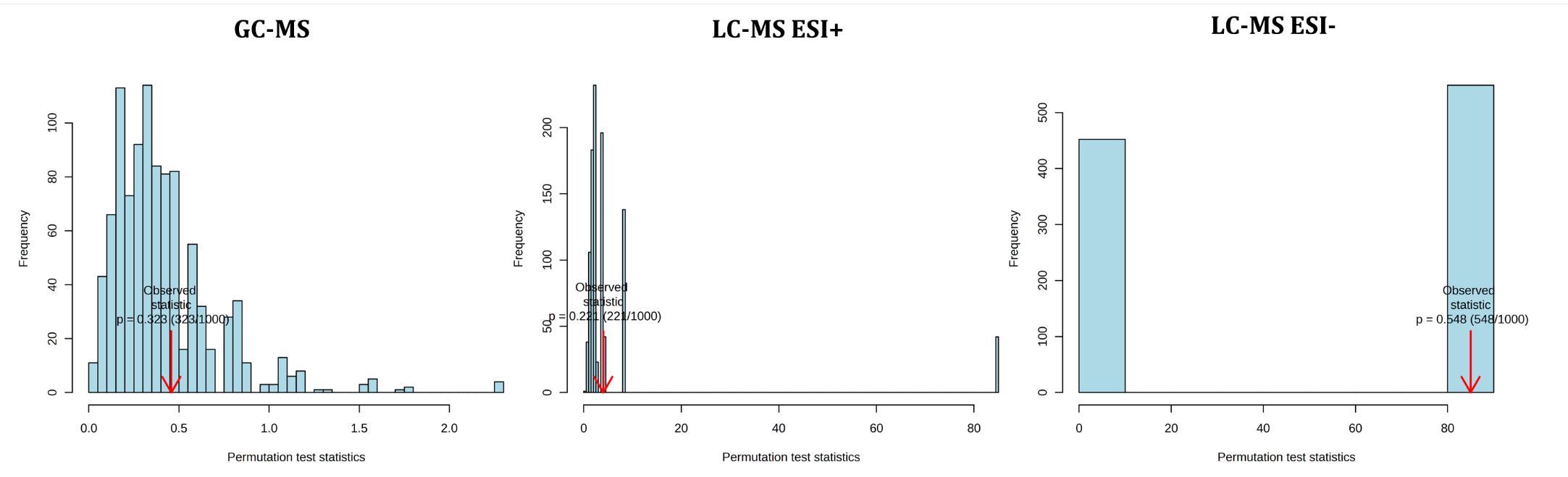


**Abbreviations:** GC-MS, gas chromatography–mass spectrometry; LC-MS, and liquid chromatography–mass spectrometry; ESI, electrospray ionization.

**Supplementary Figure 3.** Multivariate metabolomic analysis in people with HIV (PWH) on long-term suppressive ART compared by treatment. a) Partial least squares - discriminant analysis (PLS-DA); b) Permutation by separation distance (B/W) to confirm the validity of the PLS-DA models, with a permutation number of 1000.

**
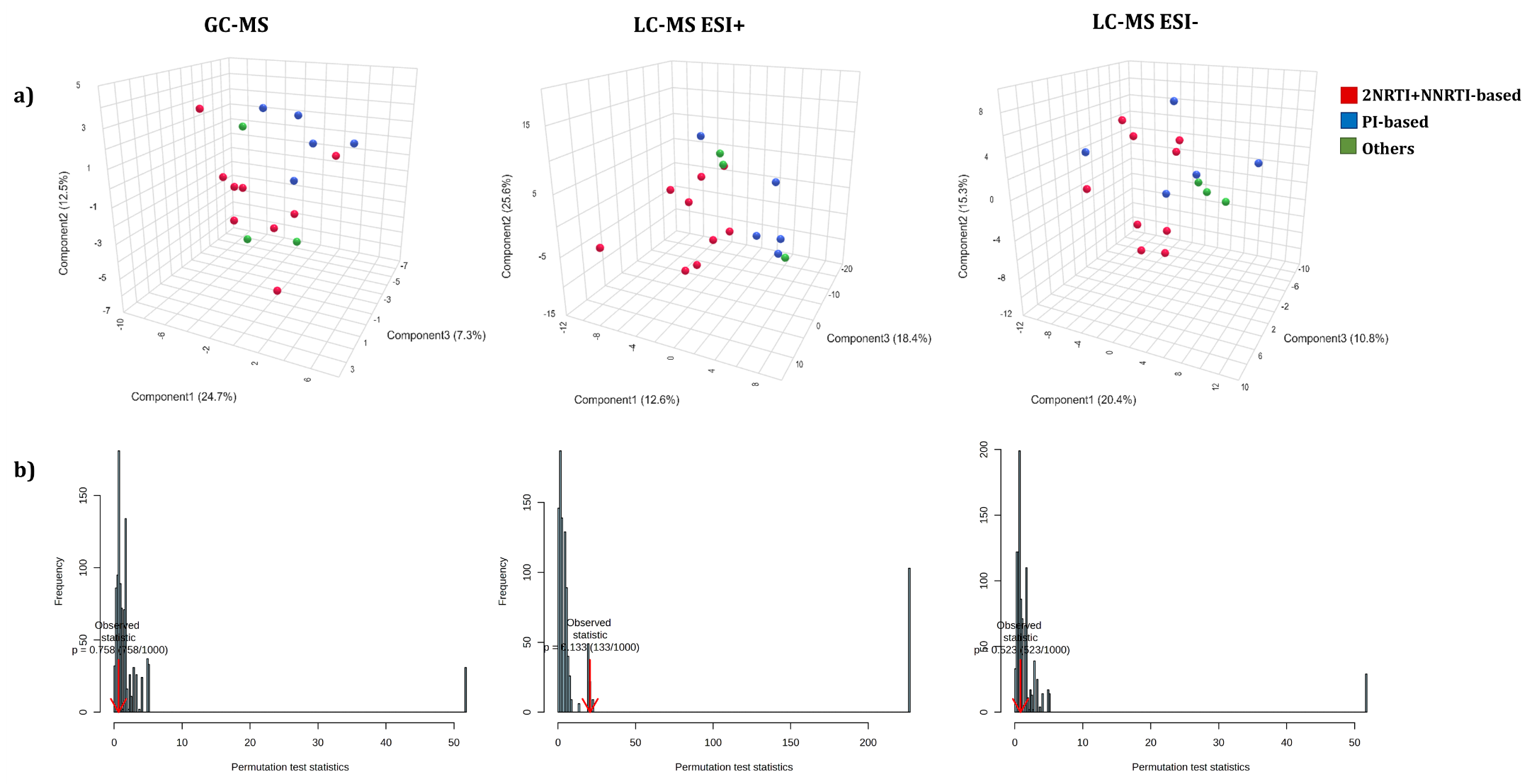
**

**Abbreviations:** GC-MS, gas chromatography–mass spectrometry; LC-MS, and liquid chromatography–mass spectrometry; ESI, electrospray ionization; NRTI, nucleoside analogue HIV reverse transcriptase inhibitor; NNRTI, non-nucleoside analogue HIV reverse transcriptase inhibitor; PI, protease inhibitor.

**Supplementary Figure 4.** Multivariate metabolomic analysis in people with HIV (PWH) on long-term suppressive ART compared by Nadir CD4+ T cell count. A) Partial least squares - discriminant analysis (PLS-DA); B) Permutation by separation distance (B/W) to confirm the validity of the PLS-DA models, with a permutation number of 1000.

**
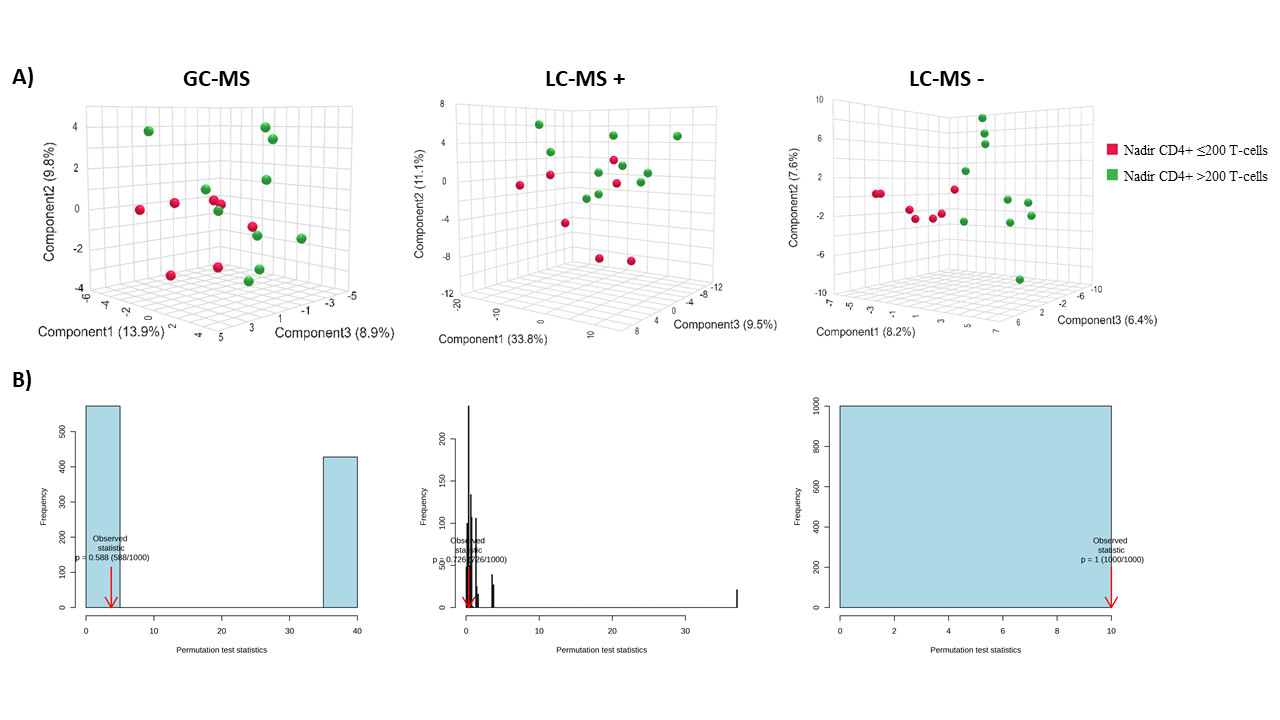
**

**Abbreviations:** GC-MS, gas chromatography–mass spectrometry; LC-MS, and liquid chromatography–mass spectrometry; ESI, electrospray ionization.

**Supplementary Figure 5.** Peak intensities of differentially significant identified features between healthy controls (HC) and people with HIV (PWH).


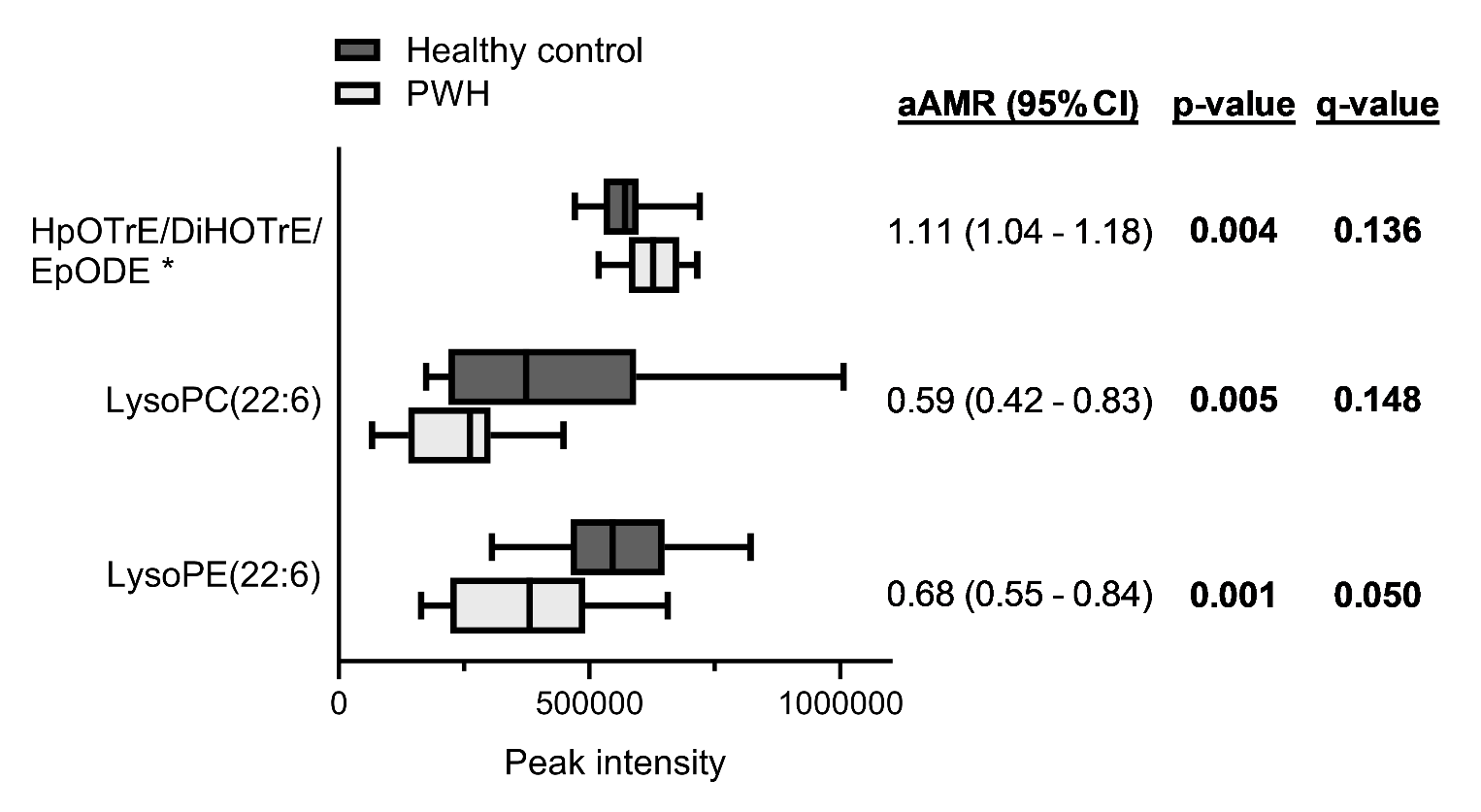


**Statistics**: Associations were calculated by Generalized Linear Model (GLM) with a gamma distribution (log-link) (dependent variable: plasma metabolites; independent variable: HIV-infection), adjusted by epidemiological characteristics (age, gender and body mass index); q-values represent p-values corrected for multiple comparisons (Benjamini and Hochberg). **Abbreviations**: aAMR, adjusted arithmetic mean ratio; 95%CI, 95% of confidence interval; p-value, level of significance; q-value, corrected level of significance; LysoPC, lysophosphocoline; LysoPE, lysophosphatidylethanolamine; HpOTrE, hydroperoxy-octadecatrienoic acid; DiHOTrE, dihydroperoxy-octadecatrienoic acid; EpODE, epoxy-octadecadienoic acid. * 12-HpOTrE / 13-HpOTrE / 13S-HpOTrE / 15,16-EpODE / 16-HpOTrE / 7,8-DiHOTrE / 9H-12(13)-EpODE / 9-HpOTrE / 9S-HpOTrE.

**Supplementary Table 1.** Plasma biomarkers concentrations in healthy controls (HC) and people with HIV (PWH).

|  | **HC** | **PWH** |  |
| --- | --- | --- | --- |
| **Plasma biomarker (pg/ml)** | **Median (IQR)** | **Median (IQR)** | ***p*-value** |
| **IL-10** | 0.48 (0.35-1.58) | 1.03 (0.78-1.75) | 0.221 |
| **TGF-β1** | 29.84 (21.72-51.46) | 45.77 (29.81-57.63) | 0.138 |
| **IL-1RA** | 154.73 (94.53-214.27) | 161.41 (137.38-219.44) | 0.296 |
| **IL-4** | 3.19 (1.76-6.63) | 3.47 (2.78-4.59) | 0.692 |
| **IP-10** | 26.90 (18.14-43.11) | 28.35 (24.61-32.61) | 0.935 |
| **MCP-1** | 14.22 (10.80-19.03) | 28.54 (16.62-39.80) | **0.017** |
| **IL-8** | 1.19 (1.19-2.16) | 1.19 (1.19-2.66) | 0.984 |
| **IL-1β** | 0.49 (0.38-1.36) | 0.81 (0.39-0.98) | 0.830 |
| **IL-18** | 96.19 (58.23-144.15) | 103.91 (52.70-132.88) | 0.935 |
| **IL-6** | 2.40 (1.65-3.67) | 3.39 (2.24-3.95) | 0.234 |
| **TNF-α** | 1.78 (0.69-3.54) | 1.93 (1.33-2.63) | 0.863 |
| **IFN-γ** | 5.25 (4.29-18.86) | 5.93 (4.01-9.03) | 0.974 |
| **IL-12p70** | 1.82 (0.95-2.96) | 2.16 (1.44-2.49) | 0.849 |
| **IL-2** | 3.57 (1.01-4.20) | 2.25 (1.01-3.88) | 0.776 |
| **IL-17A** | 1.02 (0.48-2.10) | 1..81 (1.36-2.26) | 0.077 |
| **sVCAM-1** | 215060 (115128-422438) | 309566 (205244-408810) | 0.616 |
| **sICAM-1** | 179122 (48763-680749) | 498986 (179122-889866) | 0.345 |
| **sTNFR-I** | 1484 (833.86-2236.28) | 1137 (196-2197) | 0.571 |
| **D-Dimer** | 15518 (9008-49718) | 29002 (17219-54110) | 0.333 |
| **PAI-1** | 4815 (3543-7925) | 6489 (5327-7736) | 0.109 |
| **sCD14** | 2485 (2203-3548) | 2970 (1941-6506) | 0.476 |
| **FABP2** | 0.47 (0.30-0.64) | 0.64 (0.37-1.36) | 0.129 |
| **LPS** | 1.36 (0.96-3.17) | 1.21 (0.92-1.32) | 0.419 |
| **LBP** | 0.79 (0.20-1.40) | 0.71 (0.20-1.18) | 0.749 |

**Statistics**: Values are expressed as absolute number (percentage) and median (interquartile range). P-values were calculated by Mann-Whitney tests. Abbreviations: IL, interleukin; TGF-β1, transforming growth factor beta 1; IL-1RA, IL-1 receptor antagonist; IP-10, human interferon-inducible protein 10; MCP-1, monocyte chemoattractant protein-1; TNF-α, tumor necrosis factor alpha; IFN-γ, interferon gamma; sVCAM-1, soluble vascular cell adhesion molecule-1; sICAM-1, soluble intercellular adhesion molecule-1; sTNFR-1, soluble tumour necrosis factor receptor-1; PAI-1, plasminogen activator inhibitor-1; FABP2, fatty acid-binding protein 2; LPS, lipopolysaccharide; LBP, lipopolysaccharide binding protein; IQR, interquartile range; p-value, level of significance.

**Supplementary Table 2**. Spearman correlation between significant metabolites and plasma cytokines/chemokines in people with HIV (PWH).

|  | **LysoPC(22:6)** | | | **LysoPE(22:6)** | | | **HpOTrE/DiHOTrE/EpODE*** | | |
| --- | --- | --- | --- | --- | --- | --- | --- | --- | --- |
| **Plasma biomarkers** | **rho** | **p-value** | **q-value** | **rho** | **p-value** | **q-value** | **rho** | **p-value** | **q-value** |
| IL-10 | 0.38 | 0.135 | 0.203 | 0.45 | 0.070 | 0.203 | 0.16 | 0.541 | 0.541 |
| TGF-β1 | 0.42 | 0.096 | 0.144 | 0.42 | 0.090 | 0.144 | -0.09 | 0.743 | 0.743 |
| IL-1RA | 0.26 | 0.334 | 0.761 | -0.08 | 0.761 | 0.761 | -0.13 | 0.628 | 0.761 |
| IL-4 | 0.09 | 0.725 | 0.725 | 0.31 | 0.219 | 0.658 | 0.16 | 0.528 | 0.725 |
| IP-10 | 0.00 | 0.991 | 0.991 | -0.04 | 0.897 | 0.991 | -0.04 | 0.880 | 0.991 |
| MCP-1 | **-0.52** | **0.037** | **0.112** | -0.14 | 0.602 | 0.602 | 0.41 | 0.116 | 0.174 |
| IL-8 | -0.22 | 0.417 | 0.626 | -0.26 | 0.322 | 0.626 | -0.13 | 0.644 | 0.644 |
| IL-1β | 0.41 | 0.101 | 0.302 | 0.31 | 0.221 | 0.331 | -0.12 | 0.639 | 0.639 |
| IL-18 | -0.05 | 0.863 | 0.863 | -0.23 | 0.399 | 0.598 | -0.23 | 0.387 | 0.598 |
| IL-6 | 0.29 | 0.257 | 0.386 | 0.31 | 0.225 | 0.386 | -0.08 | 0.750 | 0.750 |
| TNF-α | 0.34 | 0.175 | 0.263 | 0.44 | 0.077 | 0.230 | 0.24 | 0.347 | 0.347 |
| IFN-γ | 0.18 | 0.495 | 0.819 | 0.14 | 0.583 | 0.819 | 0.06 | 0.819 | 0.819 |
| IL-12p70 | 0.38 | 0.137 | 0.205 | 0.49 | 0.046 | 0.139 | 0.00 | 1.000 | 1.000 |
| IL-2 | 0.39 | 0.123 | 0.185 | 0.40 | 0.109 | 0.185 | 0.02 | 0.951 | 0.951 |
| IL-17A | 0.11 | 0.680 | 0.680 | 0.20 | 0.433 | 0.680 | 0.18 | 0.482 | 0.680 |
| sVCAM-1 | 0.09 | 0.743 | 0.896 | 0.03 | 0.896 | 0.896 | 0.38 | 0.138 | 0.414 |
| sICAM-1 | 0.04 | 0.888 | 0.888 | 0.04 | 0.884 | 0.888 | **0.67** | **0.004** | **0.011** |
| sTNFR-I | -0.06 | 0.827 | 0.827 | 0.07 | 0.793 | 0.827 | **0.63** | **0.009** | **0.028** |
| D-Dimer | 0.02 | 0.931 | 0.948 | -0.02 | 0.948 | 0.948 | 0.30 | 0.259 | 0.777 |
| PAI-1 | 0.11 | 0.688 | 0.688 | 0.34 | 0.192 | 0.313 | 0.33 | 0.208 | 0.313 |
| sCD14 | 0.34 | 0.188 | 0.429 | 0.27 | 0.286 | 0.429 | -0.06 | 0.823 | 0.823 |
| FABP2 | -0.19 | 0.474 | 0.580 | -0.19 | 0.462 | 0.580 | -0.14 | 0.580 | 0.580 |
| LPS | 0.17 | 0.504 | 0.504 | 0.25 | 0.333 | 0.500 | -0.28 | 0.269 | 0.500 |
| LBP | -0.32 | 0.215 | 0.432 | -0.17 | 0.505 | 0.505 | 0.27 | 0.288 | 0.432 |

**Statistics:** q-values represent p-values corrected for multiple testing using the False Discovery Rate (FDR). Those correlations with r>0.5 or r<-0.5, a significance value of p<0.05, and a q-value<0.20 are shown in bold. * 12-HpOTrE / 13-HpOTrE / 13S-HpOTrE / 15,16-EpODE / 16-HpOTrE / 7,8-DiHOTrE / 9H-12(13)-EpODE / 9-HpOTrE / 9S-HpOTrE. Abbreviations**:** p-value, level of significance; q-value, corrected level of significance; LysoPC, lysophosphocoline; LPE, lysophosphatidylethanolamine; HpOTrE, hydroperoxy-octadecatrienoic acid; DiHOTrE, dihydroperoxy-octadecatrienoic acid; EpODE, epoxy-octadecadienoic acid; IL, interleukin; TGF-β1, transforming growth factor beta 1; IL-1RA, IL-1 receptor antagonist; IP-10, human interferon-inducible protein 10; MCP-1, monocyte chemoattractant protein-1; TNF-α, tumor necrosis factor alpha; IFN-γ, interferon-gamma; sVCAM-1, soluble vascular cell adhesion molecule-1; sICAM-1, soluble intercellular adhesion molecule-1; sTNFR-1, soluble tumor necrosis factor receptor-1; PAI-1, plasminogen activator inhibitor-1; FABP2, fatty acid-binding protein 2; LPS, lipopolysaccharide; LBP, lipopolysaccharide binding protein.

**Supplementary Table 3**. Spearman correlation between significant metabolites and plasma cytokines/chemokines in healthy controls (HC).

|  | **LysoPC(22:6)** | | | **LysoPE(22:6)** | | | **HpOTrE/DiHOTrE/EpODE*** | | |
| --- | --- | --- | --- | --- | --- | --- | --- | --- | --- |
| **Plasma biomarkers** | **rho** | **p-value** | **q-value** | **rho** | **p-value** | **q-value** | **rho** | **p-value** | **q-value** |
| IL-10 | -0.19 | 0.456 | 0.456 | -0.44 | 0.080 | 0.172 | -0.40 | 0.115 | 0.172 |
| TGF-β1 | 0.02 | 0.920 | 0.920 | -0.29 | 0.232 | 0.348 | -0.31 | 0.190 | 0.348 |
| IL-1RA | -0.40 | 0.087 | 0.131 | -0.42 | 0.075 | 0.131 | -0.14 | 0.566 | 0.566 |
| IL-4 | 0.13 | 0.612 | 0.612 | -0.46 | 0.056 | 0.084 | **-0.54** | **0.021** | **0.064** |
| IP-10 | 0.37 | 0.115 | 0.115 | 0.45 | 0.052 | 0.115 | -0.38 | 0.104 | 0.115 |
| MCP-1 | **-0.67** | **0.002** | **0.005** | -0.32 | 0.188 | 0.282 | 0.24 | 0.314 | 0.314 |
| IL-8 | 0.27 | 0.263 | 0.395 | -0.18 | 0.464 | 0.464 | **-0.51** | **0.027** | **0.081** |
| IL-1β | 0.27 | 0.284 | 0.284 | -0.34 | 0.167 | 0.251 | **-0.58** | **0.011** | **0.033** |
| IL-18 | -0.41 | 0.082 | 0.224 | -0.34 | 0.149 | 0.224 | -0.04 | 0.870 | 0.870 |
| IL-6 | 0.13 | 0.619 | 0.694 | 0.10 | 0.694 | 0.694 | -0.27 | 0.291 | 0.694 |
| TNF-α | 0.08 | 0.749 | 0.749 | -0.50 | 0.042 | 0.126 | -0.41 | 0.099 | 0.148 |
| IFN-γ | -0.05 | 0.845 | 0.971 | -0.24 | 0.329 | 0.971 | -0.01 | 0.971 | 0.971 |
| IL-12p70 | 0.07 | 0.786 | 0.786 | **-0.59** | **0.012** | **0.027** | **-0.57** | **0.018** | **0.027** |
| IL-2 | 0.17 | 0.526 | 0.526 | -0.32 | 0.210 | 0.315 | -0.48 | 0.049 | 0.146 |
| IL-17A | 0.11 | 0.663 | 0.663 | **-0.50** | **0.040** | **0.119** | -0.29 | 0.266 | 0.399 |
| sVCAM-1 | 0.50 | 0.030 | 0.045 | 0.01 | 0.966 | 0.966 | **-0.55** | **0.016** | **0.045** |
| sICAM-1 | 0.39 | 0.103 | 0.155 | 0.01 | 0.974 | 0.974 | **-0.68** | **0.001** | **0.004** |
| sTNFR-I | 0.34 | 0.158 | 0.475 | -0.06 | 0.800 | 0.867 | 0.04 | 0.867 | 0.867 |
| D-Dimer | 0.06 | 0.808 | 0.915 | -0.03 | 0.915 | 0.915 | -0.21 | 0.395 | 0.915 |
| PAI-1 | 0.16 | 0.500 | 0.500 | -0.20 | 0.420 | 0.500 | -0.41 | 0.078 | 0.234 |
| sCD14 | -0.43 | 0.067 | 0.101 | **-0.66** | **0.002** | **0.007** | -0.07 | 0.786 | 0.786 |
| FABP2 | -0.47 | 0.043 | 0.129 | -0.02 | 0.932 | 0.932 | 0.17 | 0.477 | 0.716 |
| LPS | 0.19 | 0.424 | 0.521 | -0.16 | 0.521 | 0.521 | -0.26 | 0.283 | 0.521 |
| LBP | 0.01 | 0.983 | 0.983 | 0.05 | 0.829 | 0.983 | 0.19 | 0.442 | 0.983 |

**Statistics:** q-values represent p-values corrected for multiple testing using the False Discovery Rate (FDR). Those correlations with r>0.5 or r<-0.5, a significance value of p<0.05, and q-value<0.20 are shown in bold. * 12-HpOTrE / 13-HpOTrE / 13S-HpOTrE / 15,16-EpODE / 16-HpOTrE / 7,8-DiHOTrE / 9H-12(13)-EpODE / 9-HpOTrE / 9S-HpOTrE. Abbreviations: p-value, level of significance; q-value, corrected level of significance; LysoPC, lysophosphocoline; LPE, lysophosphatidylethanolamine; HpOTrE, hydroperoxy-octadecatrienoic acid; DiHOTrE, dihydroperoxy-octadecatrienoic acid; EpODE, epoxy-octadecadienoic acid; IL, interleukin; TGF-β1, transforming growth factor beta 1; IL-1RA, IL-1 receptor antagonist; IP-10, human interferon-inducible protein 10; MCP-1, monocyte chemoattractant protein-1; TNF-α, tumor necrosis factor alpha; IFN-γ, interferon-gamma; sVCAM-1, soluble vascular cell adhesion molecule-1; sICAM-1, soluble intercellular adhesion molecule-1; sTNFR-1, soluble tumor necrosis factor receptor-1; PAI-1, plasminogen activator inhibitor-1; FABP2, fatty acid-binding protein 2; LPS, lipopolysaccharide; LBP, lipopolysaccharide binding protein.
